# Redesign and reconstruction of a steviol-biosynthetic pathway for enhanced production of steviol in *Escherichia coli*

**DOI:** 10.1186/s12934-020-1291-x

**Published:** 2020-02-03

**Authors:** Jun Ho Moon, Kunjoong Lee, Jun Ho Lee, Pyung Cheon Lee

**Affiliations:** Department of Molecular Science and Technology and Department of Applied Chemistry and Biological Engineering, Ajou University, Woncheon-dong, Yeongtong-gu, Suwon, 16499 South Korea

**Keywords:** Steviol, Kaurenoic acid, Kaurene, Metabolic engineering

## Abstract

**Background:**

Steviol glycosides such as stevioside have attracted the attention of the food and beverage industry. Recently, efforts were made to produce these natural sweeteners in microorganisms using metabolic engineering. Nonetheless, the steviol titer is relatively low in metabolically engineered microorganisms, and therefore a steviol-biosynthetic pathway in heterologous microorganisms needs to be metabolically optimized. The purpose of this study was to redesign and reconstruct a steviol-biosynthetic pathway via synthetic-biology approaches in order to overproduce steviol in *Escherichia coli*.

**Results:**

A genome-engineered *E. coli* strain, which coexpressed 5′ untranslated region (UTR)-engineered geranylgeranyl diphosphate synthase, copalyl diphosphate synthase, and kaurene synthase, produced 623.6 ± 3.0 mg/L *ent*-kaurene in batch fermentation. Overexpression of 5′-UTR–engineered, N-terminally modified kaurene oxidase of *Arabidopsis thaliana* yielded 41.4 ± 5 mg/L *ent*-kaurenoic acid. Enhanced *ent*-kaurenoic acid production (50.7 ± 9.8 mg/L) was achieved by increasing the cellular NADPH/NADP^+^ ratio. The expression of a fusion protein, UtrCYP714A2-AtCPR2 derived from *A. thaliana*, where trCYP714A2 was 5′-UTR–engineered and N-terminally modified, gave 38.4 ± 1.7 mg/L steviol in batch fermentation.

**Conclusions:**

5′-UTR engineering, the fusion protein approach, and redox balancing improved the steviol titer in flask fermentation and bioreactor fermentation. The expression engineering of steviol-biosynthetic enzymes and the genome engineering described here can serve as the basis for producing terpenoids—including steviol glycosides and carotenoids—in microorganisms.

## Background

**Fig. 1:**
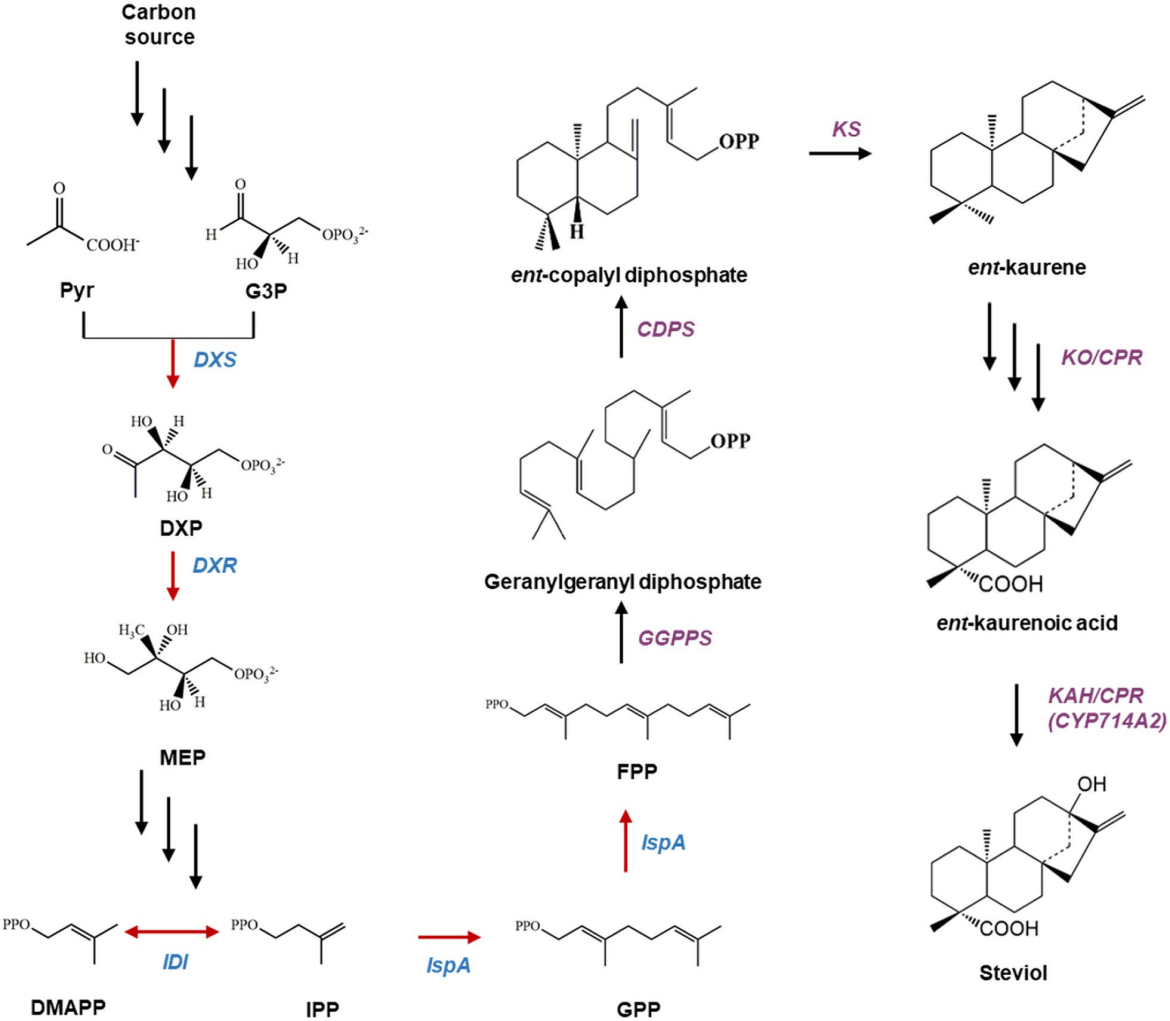
The biosynthetic pathway of steviol constructed in a heterologous host, *E. coli.* IPP, the precursor of steviol, is synthesized by the endogenous MEP pathway of *E. coli*. FPP is converted to steviol by the exogenous steviol synthesis pathway. Single arrows represent single-step reactions, while triple arrows denote multistep reactions. Red arrows indicate overexpressed genes intended to enhance the precursor pool. The genes (*dxs* from *Bacillus subtilis*, *dxr* from *E. coli*, and *idi* and *ispA* from *Enterococcus* sp.) were integrated into the genome of the MG1655 strain. CDPS, *ent*-copalyl diphosphate synthase; CPR, NADPH–cytochrome P450 reductase; DMAPP, dimethylallyl pyrophosphate; DXP, 1-deoxy-d-xylulose 5-phosphate; DXR, 1-deoxy-d-xylulose 5-phosphate reductoisomerase; DXS, 1-deoxyxylulose-5-phosphate synthase; FPP, farnesyl diphosphate; G3P, glyceraldehyde-3-phosphate; GPP, geranyl diphosphate; GGPPS, geranylgeranyl diphosphate synthase; IDI, isopentenyl diphosphate isomerase; IPP, isopentenyl pyrophosphate; IspA, farnesyl diphosphate synthase; KAH, kaurenoic acid 13-hydroxylase; KO, *ent*-kaurene oxidase; KS, *ent*-kaurene synthase; MEP, 2-C-methylerythritol 4-phosphate; Pyr, pyruvate

Steviol glycosides are diterpenoid glycosides of *ent*-kaurene present in the plant *Stevia rebaudiana* Bertoni. Given that steviol glycosides contain no calories and taste 200–300-fold sweeter than sucrose [1], these natural sweeteners may help prevent diabetes and obesity [2]. Steviol glycosides such as stevioside have therefore attracted the attention of the food and beverage industry. Steviol is biosynthesized from an isoprenoid precursor, isopentenyl pyrophosphate (IPP), which is synthesized through the methylerythritol 4-phosphate (MEP) pathway [3]. Three moles of IPP are condensed to form farnesyl diphosphate (FPP) by FPP synthase, and FPP is further condensed with one mole of IPP to form geranylgeranyl diphosphate (GGPP) by GGPP synthase (GGPPS). As shown in Fig. 1, GGPP is transformed to *ent*-copalyl diphosphate by copalyl diphosphate synthase (CDPS), and then *ent*-copalyl diphosphate is cyclized to *ent*-kaurene by kaurene synthase (KS). Finally, *ent*-kaurene is oxidized and hydroxylated by kaurene oxidase (KO) and kaurenoic acid 13-hydroxylase (KAH) with the formation of steviol [4].

Microbial production of steviol glycosides is regarded as a promising alternative to conventional methods such as extraction after open-field cultivation. Recently, efforts were made to produce *ent*-kaurene, *ent*-kaurenoic acid, steviol, and steviol glycosides in microorganisms by metabolic engineering [5, 6]. Nevertheless, the titer of steviol is relatively low in metabolically engineered microorganisms, and therefore a steviol-biosynthetic pathway in heterologous microorganisms needs to be metabolically optimized. Some studies [6] have revealed that only a small amount of *ent*-kaurenoic acid is converted in vivo to steviol (Fig. 1), suggesting that the reaction of hydroxylation of *ent*-kaurenoic acid needs to be metabolically optimized in the steviol glycoside–biosynthetic pathway. It is known that the conversion yield of *ent*-kaurenoic acid to steviol is negligible when KAH from *S. rebaudiana* (srKAH) is expressed in *Escherichia coli* [6]. Accordingly, Wang and colleagues [6] have employed CYP714A2 from *Arabidopsis thaliana* instead of srKAH and achieved 15.4 mg/L steviol in *E. coli*. In addition, the same research group has reported that 1.8 g/L *ent*-kaurene can be obtained by fed-batch fermentation (in a 5 L bioreactor) under optimized conditions.

Enzymes KO and KAH are cytochrome P450 proteins, and it is generally difficult to express functional P450 enzymes derived from plants in a heterologous bacterial system. Many plant P450 enzymes have a hydrophobic domain at the N terminus and contain transmembrane amino acid sequences, which are anchored in the endoplasmic reticulum membrane of plants [7, 8]. In addition, plant P450 enzymes need an auxiliary cytochrome P450 reductase (CPR), which transfers electrons from NADPH to P450 [9]. Plant P450 enzymes tend to be insoluble, and because the endoplasmic reticulum and CPR are not present in the heterologous *E. coli* system, functional expression of plant enzymes KO and KAH is difficult in *E. coli*. To overcome these challenges, there have been attempts to produce functional P450 proteins in *E. coli* via such approaches as N-terminal amino acid sequence modifications and optimization of electron transfer efficiency [10–12].

Plasmid expression systems are useful for the reconstruction of biosynthetic pathways and usually give a high yield of a target product. On the other hand, plasmid expression systems have some disadvantages in large-scale fermentation, e.g., segregational or structural instability and a metabolic burden [13, 14]. An expensive antibiotic supplement is necessary to maintain plasmids in host cells during the cultivation. To resolve this problem, genome engineering techniques, such as λ Red recombineering and CRISPR/Cas9, which can integrate genes into a chromosome, have been employed [15].

For higher steviol production, in the present study, our aim was to redesign a steviol-biosynthetic pathway by coexpression of enzymes GGPPS, CDPS, KS, KO, and KAH—as a modular expression unit in *E. coli*—with an engineered MEP pathway. To increase the yield of steviol, engineering of 5′ untranslated region (UTR) sequences of KS, modification of the N-terminal sequences of KO, and deletion of *gdhA*, which encodes glutamate dehydrogenase, were performed.

## Results and discussion

### Engineering the *ent*-kaurene pathway in *E. coli*

**Fig. 2:**
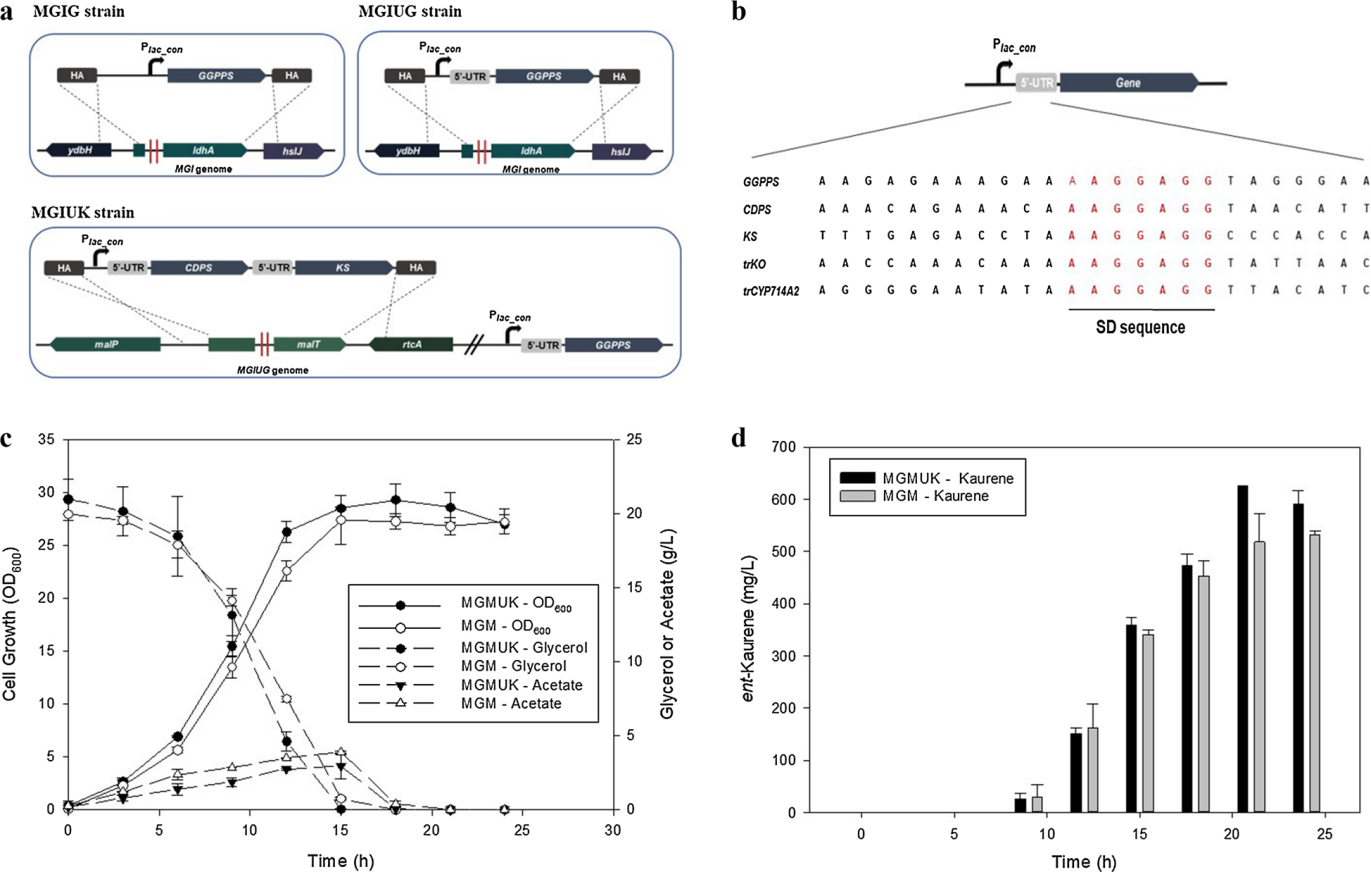
Construction of genome-edited strains expressing 5′-UTR–engineered enzymes of the pathway. Cell growth and production of *ent*-kaurene in batch bioreactor fermentation are presented too. **a** Schemes of construction of strains MGIG, MGIUG, and MGIUK. **b** Redesign of the 5′-UTRs of *GGPPS*, *CDPS*, *KS*, *trKO*, and *trCYP714A2*. **c** Cell growth of strains MGIUK and MGI/GGPPS_CDPS_KS in batch fermentation with 20 g/L glycerol as a carbon source. **d***Ent*-kaurene production by strains MGIUK and MGI/GGPPS_CDPS_KS in batch fermentation. The results represent means from three independent experiments

To investigate the effect of a GGPPS expression system (plasmid expression vs. a plasmid-free system) on the production of *ent*-kaurene, the MGI strain expressing genes *dxs*, *dxr*, *idi*, and *ispA* was chosen as a platform strain for constructing two recombinant *ent*-kaurene–producing strains (MGI/GGPPS_CDPS_KS and MGIG/CDPS_KS). The MGI strain was transformed with plasmid pSTVM_GCK, which coexpresses GGPPS, CDPS, and KS extrachromosomally (Table 2), resulting in the MGI/GGPPS_CDPS_KS strain. The genome-edited MGIG strain, which constitutively expresses GGPPS from the MGI genome (Fig. 2a), was transformed with plasmid pSTVM_CK, which coexpresses CDPS and KS extrachromosomally (Table 2), thus yielding the MGIG/CDPS_KS strain. When two *ent*-kaurene–producing strains (MGI/GGPPS_CDPS_KS and MGIG/CDPS_KS) were cultured in flasks, the MGIG/CDPS_KS strain produced 146 ± 6 mg/L *ent*-kaurene, whereas MGI/GGPPS_CDPS_KS produced 189 ± 10 mg/L *ent*-kaurene. Enhancement of the *ent*-kaurene production by GGPPS expression from the plasmid suggests that higher expression of GGPPS from the genome may increase the yield of *ent*-kaurene. Therefore, as one of the strategies for increasing GGPPS expression in the genome, engineering of the 5′-UTR was applied to GGPPS (Fig. 2b). The MGIUG strain expressing 5′-UTR_GGPPS in the MGI genome was constructed and transformed with pSTVM_CK. When the MGIUG/CDPS_KS strain was cultured in flasks, the production of *ent*-kaurene reached 195 ± 9 mg/L, which was comparable to the 189 ± 10 mg/L obtained with GGPP expressed by a plasmid expression system (MGI/GGPPS_CDPS_KS).

Next, because the 5′-UTR–engineered GGPPS successfully increased the production of *ent*-kaurene, 5′-UTR–engineered genes *CDPS* and *KS* were constructed and further modified to be expressed from the genome of the MGIU strain (named MGIUK). When the production of *ent*-kaurene in the MGIUK strain was compared with that of MGI/GGPPS_CDPS_KS, which coexpresses GGPPS, CDPS, and KS from a plasmid, the MGIUK strain produced 205 ± 35 mg/L *ent*-kaurene, whereas MGI/GGPPS_CDPS_KS produced 191 ± 7 mg/L in flasks. This result indicates that the enzymes of the 5′-UTR–engineered *ent*-kaurene pathway when expressed from the genome were comparably functional as compared to the plasmid-based *ent*-kaurene pathway system developed in this study. The production of *ent*-kaurene (205 ± 35 mg/L) in flask fermentation by the genome-engineered MGIUK strain was also higher than the previously reported titers of 194.12 mg/L [6] and 179.6 mg/L [5] in an inducible or constitutive plasmid expression system. Notably, the growth of MGIUK was faster than that of MGI/GGPPS_CDPS_KS (OD_600_ of 23 ± 4.5 vs. 20 ± 4.1 at 48 h of culture). This result suggests that a plasmid-free system may save cellular energy and building blocks that are used to maintain and replicate the plasmids. To further investigate the growth and *ent*-kaurene production by the MGIUK strain, batch bioreactor fermentation was carried out with 20 g/L glycerol as a carbon source (Fig. 2c). The MGIUK strain produced 623.6 ± 3.0 mg/L *ent*-kaurene, which was 11% higher than the 531.5 ± 7.1 mg/L *ent*-kaurene generated by MGI/GGPPS_CDPS_KS after 21 h of batch bioreactor fermentation (Fig. 2d). As observed in the flask fermentation, *ent*-kaurene production of the MGIUK strain was higher than the previously reported titer of 578 mg/L obtained via a constitutive plasmid expression system in batch fermentation [5]. The highest concentration of *ent*-kaurene reported so far is 1.8 g/L, obtained with an inducible plasmid in a fed-batch fermentation system [6]. Even though a direct comparison between our data and the results from Ref. [6] is problematic due to different fermentation modes (batch vs. fed-batch), the productivity of the MGIUK strain was better than that of the previously reported strain: 623.6 ± 3.0 mg/L *ent*-kaurene after 21 h cultivation (Fig. 2d) vs. ~ 600 mg/L *ent*-kaurene after 63 h cultivation [6]. The growth of MGIUK reached a maximum OD_600_ of 31, whereas the growth of strain MGI/GGPPS_CDPS_KS reached a maximum OD_600_ of 28 at 18 h of culture. This observation confirms that the plasmid-free *ent*-kaurene-producing MGIUK strain is comparable with the plasmid-based *ent*-kaurene host, MGI/GGPPS_CDPS_KS.

### Engineering the *ent*-kaurenoic acid pathway in *E. coli*

**Fig. 3:**
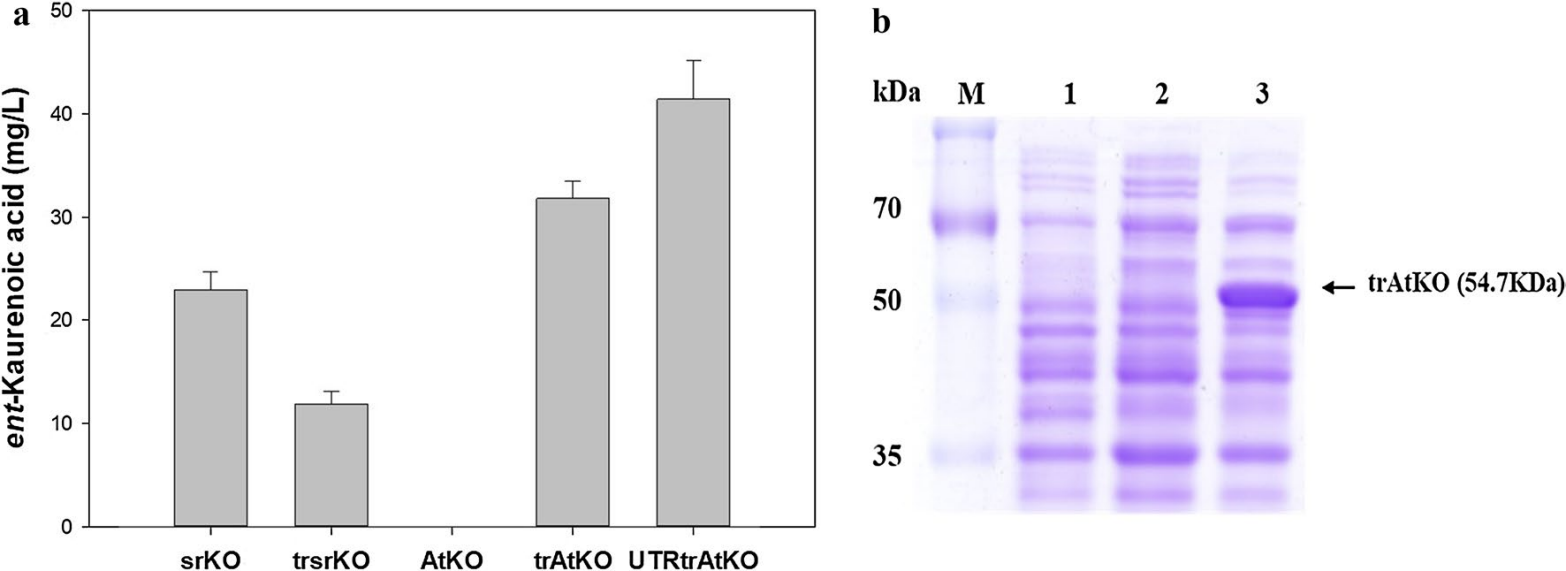
Functional complementation of native KOs and N-terminally engineered KOs of *A. thaliana* and *S. rebaudiana.* SDS-PAGE analysis of the expression of these KOs is presented too. **a** The production of *ent*-kaurenoic acid in the MGIUK strains expressing SrKO, trSrKO, AtKO, trAtKO, and UtrAtKO. The results represent means from five independent experiments. **b** SDS-PAGE analysis [in a 10% (w/v) gel] of a crude protein extract of *E. coli* BL21 (DE3) harboring pET21α (+), pET21α (+)_AtKO, and pET21α (+)_trAtKO. M: protein molecular weight markers, lane 1: pET21α (+), lane 2: pET21α (+)_AtKO, and lane 3: pET21α (+)_trAtKO

The pathway extension from *ent*-kaurene to *ent*-kaurenoic acid requires the KO enzyme and its electron transfer partner CPR. So far, two KOs (SrKO from *S. rebaudiana* and AtKO from *A. thaliana* [16]) have been mainly used for the reconstruction of the steviol and steviol glycoside pathway in microorganisms. To enhance the expression of SrKO and AtKO in the heterologous host (*E. coli)*, the N-terminal amino acid sequences of the two KOs were truncated and fused with the previously studied “MALLLAVF” sequence [17–19], thus resulting in trSrKO and trAtKO, respectively. CPR from *A. thaliana* (named AtCPR2) [6] served as an electron transfer partner for trSrKO and trAtKO in *E. coli*. During cultivation in flasks, the expression of trSrKO_ AtCPR2 yielded less *ent*-kaurenoic acid (13.5 ± 1.4 mg/L) as compared to native SrKO_AtCPR2 (22.9 ± 1.7 mg/L) in the MGIUK strain. By contrast, trAtKO_AtCPR2 produced up to 31.8 ± 1.7 mg/L *ent*-kaurenoic acid, whereas native AtKO_AtCPR2 did not produce *ent*-kaurenoic acid in the MGIUK strain (Fig. 3a). SDS-PAGE analysis confirmed that native AtKO was not expressed, which might explain why no production of *ent*-kaurenoic acid was observed when AtKO was used, while trAtKO was highly expressed in *E. coli* BL21 (DE3) (Fig. 3b). Given that trAtKO produced 40% more *ent*-kaurenoic acid than did native SrKO (31.8 ± 1.7 vs. 22.9 ± 1.7 mg/L), further engineering of the 5′-UTR of trAtKO was carried out, and this gene was then expressed in the MGIUK strain. As expected, 5′-UTR–engineered trAtKO (named UtrAtKO) significantly increased *ent*-kaurenoic acid production up to 41.4 ± 5 mg/L compared to 31.8 ± 1.7 mg/L obtained by the expression of trAtKO in the MGIUK strain (Fig. 3a).

### The effect of the NADPH/NADP^+^ ratio on the production of *ent*-kaurenoic acid

It has been well documented that the cellular redox balance and cofactor availability significantly affect the yield of metabolites in microorganisms [20–22]. NADPH acts as a redox cofactor for KO in the steviol pathway. Therefore, because a higher NADPH/NADP^+^ ratio increased the production of *ent*-kaurenoic acid, a MGIUKN strain was constructed by deletion of the *gdhA* gene encoding glutamate dehydrogenase [21] in the MGIUK strain. As expected, the NADPH/NADP^+^ ratio (0.51 ± 0.04) in the MGIUKN strain was 10% higher than that in MGIUK (0.44 ± 0.01). The impact of the higher NADPH/NADP^+^ ratio on the production of *ent*-kaurenoic acid was investigated through flask fermentation, by cultivation of MGIUKN/UtrAtKO_AtCPR2, with MGIUK/UtrAtKO_AtCPR2 serving as a control. MGIUKN/UtrAtKO_AtCPR2 produced 22.5% higher *ent*-kaurenoic acid concentration than did MGIUK/UtrAtKO_AtCPR2 (50.7 ± 9.8 vs. 41.4 ± 5 mg/L), suggesting that the cellular redox balance is important for the conversion of *ent*-kaurene to *ent*-kaurenoic acid.

### Engineering the steviol pathway in *E. coli*

**Fig. 4:**
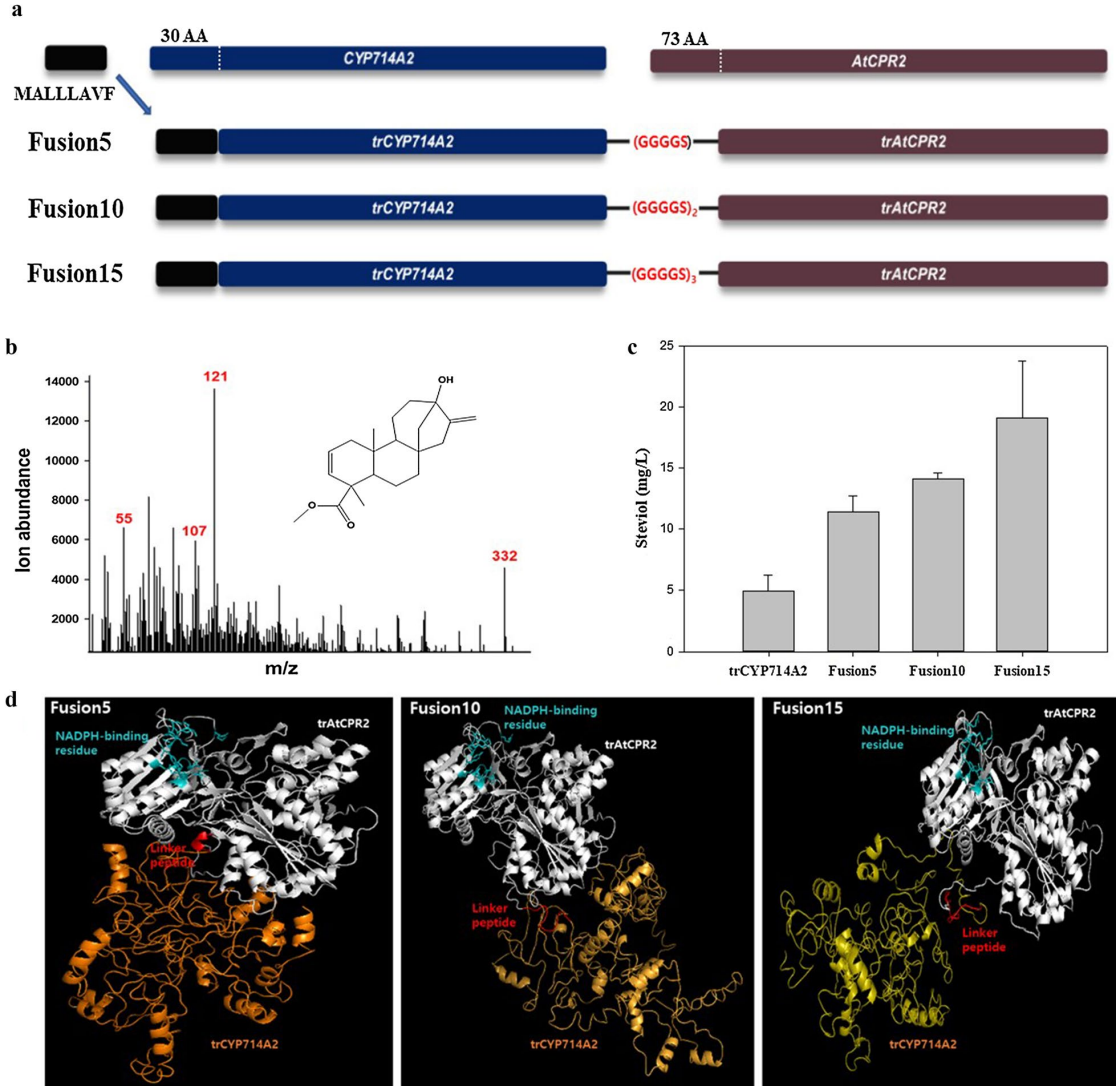
The design of fusion versions of CYP714A2-AtCPR2. The impact of the expression of the fusion proteins on steviol production is illustrated too. **a** Schematic representation of the design of the three fusion proteins (Fusion 5, Fusion 10, and Fusion 15) with three linker peptides of different lengths (GGGGS)_n=1–3_. **b** The GC–MS spectrum of steviol methyl ester that was generated after methylation of steviol produced by fermentation. **c** The effect of the expression of three fusion proteins on steviol production by the MGIUKN strain in flask fermentation reactions. The non-fusion form of UtrCYP714A2_AtCPR2 served as a control. The results represent means from five independent experiments. **d** Homology models of *A. thaliana* trCYP714A2 fused to trAtCPR2. TrAtCPR2 is white, trCYP714A2 is orange, the linker peptides are red, and NADPH-binding residues are cyan

**Fig. 5:**
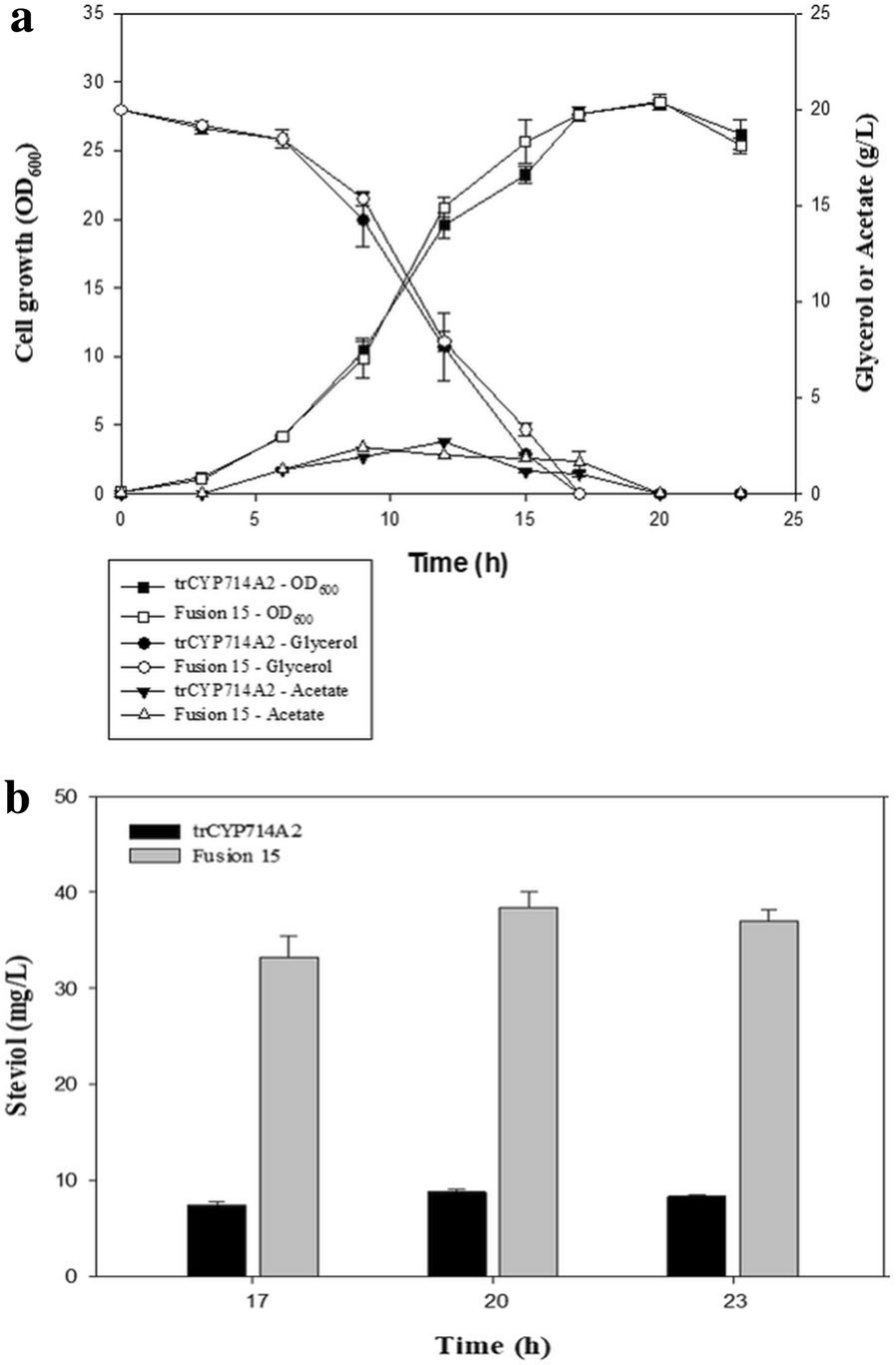
Cell growth and production of steviol in batch bioreactor fermentation. **a** Cell growth of strains MGIUKN/UtrAtKO_UtrCYP714A2_AtCPR2 and MGIUKN/UtrAtKO_Fusion15_AtCPR2 in batch fermentation with 20 g/L glycerol as a carbon source. **b** Steviol production of strains UtrAtKO_UtrCYP714A2_AtCPR2 and MGIUKN/UtrAtKO_Fusion15_AtCPR2 in batch fermentation. The results represent means from three independent experiments

The pathway extension from *ent*-kaurenoic acid to steviol was implemented via expression of CYP714A2 of *A. thaliana* instead of KAH of *S. rebaudiana* because CYP714A2 shows better performance than KAH does [6]. Likewise, to construct UtrAtKO, 5′-UTR–engineered trCYP714A2 (named UtrCYP714A2) was constructed (Fig. 4a) and was coexpressed with UtrAtKO_AtCPR2 in strains MGIUKN and MGIUK. Similarly to the enhanced *ent*-kaurenoic acid production in the MGIUKN strain, MGIUKN/UtrAtKO_UtrCYP714A2_AtCPR2 produced more steviol (Fig. 4b) than MGIUK/UtrAtKO_UtrCYP714A2_AtCPR2 did in flask cultures (5.0 ± 0.2 vs. 4.2 ± 1.1 mg/L). As one of the strategies used to increase steviol production, the electron transfer between UtrCYP714A2 and AtCPR2 was improved by fusing them through a linker peptide. UtrCYP714A2 was structurally linked to trAtCPR2 (from which 72 amino acid residues were deleted at the N terminus) through one of three peptide linkers (GGGGS)_n=1–3_. The presence and length of a flexible linker (GGGGS)_n=1–3_ significantly influenced steviol production in the MGIUKN strain. In comparison with the 5.0 ± 0.2 mg/L steviol obtained by the expression of UtrCYP714A2_AtCPR2 without the fusion, the highest concentration of steviol, 19.1 ± 4.6 mg/L, was produced when fusion 15 [(GGGGS)_n=3_] was expressed, followed by 14.1 ± 0.5 mg/L [fusion 10, (GGGGS)_n=2_] and 11.4 ± 1.3 mg/L [fusion 5, (GGGGS)_n=1_; Fig. 4c]. These results and homology of the models of the three fusion proteins (Fig. 4c) mean that greater linker length does not interfere with the interaction of the two proteins (sterically) and increases the efficiency of electron transfer. The 19.1 ± 4.6 mg/L steviol concentration yielded by fusion 15 was greater than a previously reported concentration, 15.47 mg/L [6]. To further investigate the growth and steviol production, batch bioreactor fermentation was performed with 20 g/L glycerol as a carbon source for the MGIUKN strain expressing either UtrAtKO_Fusion15_AtCPR2 or UtrAtKO_UtrCYP714A2_AtCPR2. The growth of the two strains was similar, and reached maximum OD_600_ values of 28.6 and 28.1, respectively (Fig. 5a). MGIUKN/UtrAtKO_Fusion15_AtCPR2 produced the highest concentration of steviol (38.4 ± 1.7 mg/L) at 20 h of culture, which was 4.3-fold greater than the 8.8 ± 0.3 mg/L concentration generated by the control MGIUKN/UtrAtKO_UtrCYP714A2_AtCPR2 strain (Fig. 5b). Because our steviol titer (38.4 ± 1.7 mg/L) was obtained under suboptimal fermentation conditions, a future study on fermentation optimization, e.g., by means of medium components, may further raise the steviol titer.

## Conclusions

In this study, a heterologous steviol-biosynthetic pathway was redesigned and reconstructed via a synthetic-biology approach to overproduce steviol in *E. coli*. 5′-UTR engineering, the fusion protein approach, and redox balancing improved the steviol titer in flask fermentation and bioreactor fermentation. The expression engineering of steviol-biosynthetic enzymes and genome engineering described here can serve as a basis for the production of terpenoids, including carotenoids, in microorganisms. Synthetic biology and metabolic engineering can be directly applied to the pathway engineering of steviol glycosides, which have a high value in the food industry.

## Methods

### Strains, plasmids, and culture conditions

All strains and plasmids used in this study are listed in Table 1. Plasmids pMP11 and pgRNA were provided by the Technical University of Denmark. For gene cloning, *E. coli* Top10 was cultivated in the Luria–Bertani (LB) medium (10 g/L tryptone, 5 g/L yeast extract, and 5 g/L NaCl) at 37 °C with shaking at 250 g. For the preparation of steviol pathway products, recombinant *E. coli* strains were grown at 30 °C with shaking at 250 g in 500 mL flasks with the Terrific Broth (TB) medium (12 g/L tryptone, 24 g/L yeast extract, 0.17 M KH_2_PO_4_, and 0.72 M K_2_HPO_4_) supplemented with 10 g/L glycerol. In particular, when the *E. coli* strain producing *ent*-kaurene was cultured, 20% (v/v) *n*-dodecane was overlaid on the TB medium. Ampicillin (100 μg/mL) and chloramphenicol (50 μg/mL) were added as required.

**Table 1:** Strains and plasmids used in this study

Strains or plasmids	Relevant properties	Source
Strains		
TOP10	F^−^*mcr*A Δ(*mrr*-*hsd*RMS-*mcr*BC) φ80*lac*ZΔM15 Δ*lac*X74 *rec*A1 *ara*D139 Δ(*ara*-*leu*)7697 *gal*U *gal*K *rps*L (Str^R^) *end*A1 *nup*G	Invitrogen
BL21 (DE3)	F^−^*omp*T *gal dcm lon hsd*S_B_(*r*_*B*_^−^*m*_*B*_^−^) λ(DE3 [*lac*I *lac*UV*5*-T*7p07 ind*1 *sam*7 *nin*5]) [*mal*B^+^]_K-12_(λ^S^)	NEB
MG1655	F^−^, λ^−^, *rph*-*1*	KCTC
MGI	MG1655*△ilvG::dxs△glvC::idi△yjbI::ispA△agaAV::dxr*	unpublished
MGIG	MGI*△ldhA::GGPPS*	This study
MGIUG	MGI*△ldhA::*UTR *GGPPS*	This study
MGIUK	MGIUE*△malT::*UTR*CDPSKS*	This study
MGIUKN	MGIUK*△gdhA*	This study
Plasmids		
pUCM	Cloning vector derived from pUC19; constitutive *lac* promoter, Amp^R^	[25]
pUCM_GGPPS	ColE1 ori, constitutive *lac* promoter, expressing GGPPS, Amp^R^	[5]
pUCM_CDPS	ColE1 ori, constitutive *lac* promoter, expressing CDPS, Amp^R^	[5]
pUCM_KS	ColE1 ori, constitutive *lac* promoter, expressing KS, Amp^R^	[5]
pUCM_GGPPS(U)	ColE1 ori, constitutive *lac* promoter, expressing GGPPS, synthetic 5′-UTR, Amp^R^	This study
pUCM_CDPS(U)	ColE1 ori, constitutive *lac* promoter, expressing CDPS, synthetic 5′-UTR, Amp^R^	This study
pUCM_KS(U)	ColE1 ori, constitutive *lac* promoter, expressing KS, synthetic 5′-UTR, Amp^R^	This study
pSTVM2	Cloning vector derived from pSTV29; constitutive *lac* promoter, Cm^R^	This study
pSTVM_CK	p15A ori, constitutive *lac* promoter, expressing CDPS and KS, Cm^R^	This study
pSTVM_GCK	p15A ori, constitutive *lac* promoter, expressing GGPPS, CDPS, and KS, Cm^R^	This study
pSTVM_GCK(U)	p15A ori, constitutive *lac* promoter, expressing GGPPS, CDPS and KS, synthetic 5′-UTR, Cm^R^	This study
pUCrop	ColE1 ori, rop, Amp^R^	This study
pUCrop_AtKO	ColE1 ori, rop, constitutive *lac* promoter, expressing AtKO, Amp^R^	This study
pUCrop_AtCPR2	ColE1 ori, rop, constitutive *lac* promoter, expressing AtCPR2, Amp^R^	This study
pUCrop_trAtKO	ColE1 ori, rop, constitutive *lac* promoter, expressing modified AtKO, Amp^R^	This study
pUCrop_trAtKO (U)	ColE1 ori, rop, constitutive *lac* promoter, expressing modified AtKO, synthetic 5′-UTR, Amp^R^	This study
pUCrop_AtKO_AtCPR2	ColE1 ori, rop, constitutive *lac* promoter, expressing AtKO and AtCPR2, Amp^R^	This study
pUCrop_trAtKO_AtCPR2	ColE1 ori, rop, constitutive *lac* promoter, expressing modified AtKO and AtCPR2, Amp^R^	This study
pUCrop_SrKO_AtCPR2	ColE1 ori, rop, constitutive *lac* promoter, expressing SrKO and AtCPR2, Amp^R^	This study
pUCrop_trSrKO_AtCPR2	ColE1 ori, rop, constitutive *lac* promoter, expressing modified SrKO and AtCPR2, Amp^R^	This study
pUCrop_trAtKO(U)_AtCPR2	ColE1 ori, rop, constitutive *lac* promoter, expressing modified AtKO and AtCPR2, synthetic 5′-UTR, Amp^R^	This study
pUCrop_trCYP714A2(U)	ColE1 ori, rop, constitutive *lac* promoter, expressing modified CYP714A2, synthetic 5′-UTR, Amp^R^	This study
pUCrop_Fusion5(U)	ColE1 ori, rop, constitutive *lac* promoter, expressing modified CYP714A2 and AtCPR2, chimera, synthetic 5′-UTR, Amp^R^	This study
pUCrop_Fusion10(U)	ColE1 ori, rop, constitutive *lac* promoter, expressing modified CYP714A2 and AtCPR2, chimera, synthetic 5′-UTR, Amp^R^	This study
pUCrop_Fusion15(U)	ColE1 ori, rop, constitutive *lac* promoter, expressing modified CYP714A2 and AtCPR2, chimera, synthetic 5′-UTR, Amp^R^	This study
pUCrop_trAtKO(U)_AtCPR2_trCYP714A2(U)	ColE1 ori, rop, constitutive *lac* promoter, expressing modified AtKO, AtCPR2 and modified CYP714A2, synthetic 5′-UTR, Amp^R^	This study
pUCrop_trAtKO(U)_AtCPR2_Fusion5(U)	ColE1 ori, rop, constitutive *lac* promoter, expressing modified AtKO, AtCPR2 and fusion protein, synthetic 5′-UTR, Amp^R^	This study
pUCrop_trAtKO(U)_AtCPR2_Fusion10(U)	ColE1 ori, rop, constitutive *lac* promoter, expressing modified AtKO, AtCPR2 and fusion protein, synthetic 5′-UTR, Amp^R^	This study
pUCrop_trAtKO(U)_AtCPR2_Fusion15(U)	ColE1 ori, rop, constitutive *lac* promoter, expressing modified AtKO, AtCPR2 and fusion protein, synthetic 5′-UTR, Amp^R^	This study
pET21α(+)	f1 ori, T7 promoter, C-terminal His-tag sequence, Amp^R^	Novagen
pET21α(+)_AtKO	f1 ori, T7 promoter, inducible expression of His_6_-tagged AtKO, Amp^R^	This study
pET21α(+)_trAtKO	f1 ori, T7 promoter, inducible expression of His_6_-tagged modified AtKO, Amp^R^	This study
pMP11	pKD46 with constitutively expressed Cas9, aTc gRNA targeting ColE1 origin, Amp^R^	[27]
pgRNA	Constitutively expressed sgRNA	[27]
pgRNA_ldhA	Constitutively expressed sgRNA targeting ldhA, ColE1 ori, Cm^R^	This study
pgRNA_malT	Constitutively expressed sgRNA targeting gdhA, ColE1 ori, Cm^R^	This study
pgRNA_gdhA	Constitutively expressed sgRNA targeting malT, ColE1 ori, Cm^R^	This study

### Construction of plasmids

The primer sequences used for the construction of the plasmids are listed in Table 2. To obtain the optimized 5′-UTRs of GGPPS, CDPS, KS, trAtKO, and trCYP714A2, UTR designer [23] (http://sbi.postech.ac.kr/utr_designer) was utilized, and the optimized sequences of the 5′-UTRs are listed in Table 2. Each optimized 5′-UTR was fused to the corresponding gene by polymerase chain reaction (PCR), with gene-specific primers containing the 5′-UTR sequence and then was cloned into vectors pUCM [24] and pUCrop by Gibson assembly [25]. Three plasmids expressing GGPPS, CDPS, or KS (Table 1) served as templates for cloning the genes encoding GGPPS, CDPS, and KS.

**Table 2:** Primers and other oligonucleotides used in this study

Name	Sequence (5′ → 3′)^a,b^
Primers for cloning	
pUC_AtKO_F	gcTCTAGAaggaggattacaaaatggccttcttctccatga
pUC_AtKO_R	ataagaatGCGGCCGCttaagaacgccttggattga
pUC_AtCPR2_F	gcTCTAGAaggaggattacaaaatgtcctcttcttcttcttc
pUC_AtCPR2_R	ataagaatGCGGCCGCttaccatacatctctaagatatc
pUC_CYP714A2_F	gcTCTAGAaggaggattacaaaatggagagcctggtggtc
pUC_CYP714A2_R	tcccCCCGGGttagacgacacggatcacga
pUC_trAtKO_F	gcTCTAGAaggaggattacaaaatggctttacttctggcagtttttaagaaacttctctccttctc
pUC_trCYP714A2_F	gcTCTAGAaggaggattacaaaatggctttacttctggcagtttttcgtgcggttgtcgagcag
pET_AtKO_F	cgGGATCCcatggccttcttctccatga
pET_AtKO_R	ccgCTCGAGagaacgccttggattgataat
pET_trAtKO_F	cgGGATCCatggctttacttctggcagt
Primers for Gibson assembly	
gibson_pV_F	ggccgctgcggtattttc
gibson_pV_GGPPS_R	ttccctacctccttttctttctcttcgctcacaattccacacaa
gibson_pV_CDPS_R	aatgttacctcctttgtttctgtttcgctcacaattccacacaa
gibson_pV_KS_R	tggtgggcctcctttaggtctcaaacgctcacaattccacacaa
gibson_pV_trAtKO_R	gttaatacctcctttttgtttggttcgctcacaattccacacaa
gibson_pV_trCYP714A2_R	gatgtaacctcctttatattcccctcgctcacaattccacacaa
gibson_GGPPS_F	tgagcgaagagaaagaaaaggaggtagggaaatggcgtttgaacagcgg
gibson_CDPS_F	tgagcgaaacagaaacaaaggaggtaacattatgaagaccggcttcatct
gibson_KS_F	tgagcgtttgagacctaaaggaggcccaccaatgaatctttcactatgcatc
gibson_trAtKO_F	tgagcgaaccaaacaaaaaggaggtattaacatggctttacttctggcagt
gibson_trCYP714A2_F	tgagcaggggaatataaaggaggttacatcatggctttacttctggcag
gibson_insert_R	aataccgcacagatgcgtaa
gibson_Fusion5_AtCPR2_F	ggtggcggcggaagcaggagatccggttctgg
gibson_Fusion5_trCYP714A2_R	cggatctcctgcttccgccgccaccgacgacacggatcacgac
gibson_Fusion10_AtCPR2_F	gtggcggtagtggcggtggtggaagtaggagatccggttctgg
gibson_Fusion10_trCYP714A2_R	cttccaccaccgccactaccgccaccaccgacgacacggatcacgac
gibson_Fusion15_AtCPR2_F	caggtggtgggggatctggtggcggtggcagtaggagatccggttctgg
gibson_Fusion15_trCYP714A2_R	ccgccaccagatcccccaccacctgacccccctcctccgacgacacggatcacgac
Primers for subcloning	
pSTVM2-sub-USER-3-F	agacagucataagtgcgg
pSTVM2-sub-USER-1-R	atgcaacucgtaggacag
pUC- sub-USER-3-F	agacagucaatctgctctgatgcc
pUC- sub-USER-1-R	atgcaacuca taatgaatcggccaac
pUC-sub-USER-1-F	agttgcaucccgactggaaagcg
pUC-sub-USER-2-F	atccatgucccgactggaaagcg
pUC-sub-USER-5-F	atatgcgaucccgactggaaagcg
pUC-sub-USER-2-R	acatggauatgcggtgtgaaatacc
pUC-sub-USER-5-R	atcgcatauatgcggtgtgaaataccg
pUC-sub-USER-3-R	actgtcuatgcggtgtgaaataccg
Primers for genome editing	
ldhA_up_F	aaacctttacgcgtaatgcg
ldhA_up_R	ctttccagtcgtgctataaacggcgagtt
GGPPS_F	tttatagcacgactggaaagcgggcag
GGPPS_R	gcaagattaaagaaaataccgcagcggcc
ldhA_Down_F	ggtattttctttaatcttgccgctcccc
ldhA_Down_R	ggttagcgcacatcatacg
malT_up_F	aaaaatggccgttgcgtatt
malT_up_R	tttccagtcgggacatggatagttaatcacttcactgtgga
CDPS_F	atccatgtcccgactggaaagcg
CDPS_R	tcatattacaatctcgaacacc
KS_F	tgttcgagattgtaatatgatttgagacctaaaggaggc
KS_R	actgtctatgcggtgtgaaataccg
malT_Down_F	tttcacaccgcatagacagtcaattgctgaagatgatggg
malT_Down_R	gccgggtaataccgtctc
Confirm_GGPPS_F	cggattgaagcggcaatg
Confirm _GGPPS_R	tgcccagcgtctcggcat
Confirm _CDPS/KS_F	aactcatcctcaataccaac
Confirm _CDPS/KS_R	ggctacccatgctgtgtc
Confirm _gdhA_F	ttatggctttacgcgccgc
Confirm _gdhA_R	gggacaattgaagaagaact
Oligonucleotides for deletion of *gdhA*	
gdhA-MAGE	aatatataagggttttatatctatggatcagacatattctctggcgcagggtgtgatttaagttgtaaatgcctgatggc

^a^Capital letters indicate restriction sites

^b^Underlining denotes 5′-UTR sequences

To clone the genes encoding CYP714A2, AtKO, and AtCPR2 from *A. thaliana*, stem cells of *A. thaliana* were ground into a powder with a pestle in liquid nitrogen. Total RNA was extracted from the powder with the easy-BLUE™ RNA Extraction Kit (iNtRON Biotech, Korea) and then treated with DNase I (TaKaRa, Japan) at 37 °C for 30 min. After inactivation of DNase I, 1 μg of total RNA was subjected to cDNA synthesis using the ReverTraAce qPCR RT Kits (Toyobo, Japan). Genes encoding CYP714A2, AtKO, and AtCPR2 were amplified by PCR with gene-specific primers (Table 2). The three amplified genes were cloned into pUCrop, resulting in plasmids pUCrop_CYP714A2, pUCrop_AtKO, and pUCrop_AtCPR2. Assembly of two or three genes in one vector system, e.g., pUCrop_AtKO_AtCPR2 (Table 1), was carried out via the uracil excision cloning technology (USER) [26].

### Construction of an *ent*-kaurene pathway in the genome of *E. coli*

The *E. coli* MG1655 strain expressing genes *dxs*, *dxr*, *idi*, and *isp*A (Table 1) served as a platform strain (named MGI) for engineering the *ent*-kaurene pathway. An MGIG strain expressing GGPPS was constructed by integration of a synthetic module expressing GGPPS into an *ldhA* site in the MGI genome. Similarly, an MGIUG strain was constructed by integration of a synthetic module expressing 5′-UTR_GGPPS into an *ldhA* site in the MGI genome. An MGIUK strain was created via integration of a synthetic module expressing 5′-UTR_CDPS and 5′-UTR_KS into a *malT* site in the MGIUG genome. The above-mentioned genome integration procedures were performed by CRISPR/Cas9 genome editing [27]. Linear donor DNA fragments containing combined 400 bp homology arm sequences were constructed by overlap extension PCR with gene-specific primers (Table 2). To construct two guide RNA (gRNA) vectors (pgRNA_ldhA and pgRNA_malT), the pgRNA plasmid backbone was amplified by PCR with primers containing 20 bp of a target-specific gRNA sequence. To improve the cutting efficiency of Cas9, the gRNA sequences were designed in the CHOPHOP software (http://chopchop.cbu.uib.no/). MGIG, MGIUG, and MGIUK were selected by colony PCR, and the sequences of the edited genome sites of the three strains were verified by Sanger sequencing (Macrogen, Korea). The three genome-edited strains were cured of the plasmids by the addition of 200 ng/ml anhydrotetracycline and incubation at 37 °C.

### Construction and expression of N-terminal mutant AtKO (trAtKO)

The transmembrane region of AtKO was predicted by means of the online TMHMM software (http://www.cbs.dtu.dk/services/TMHMM/). The predicted region, consisting of 24 amino acid residues of AtKO, was replaced with an 8-mer peptide (MALLLAVF; derived from 17α bovine hydroxylase [6]), by PCR with primers (Table 2). The resulting N-terminally modified trAtKO was cloned into the pET21α(+) vector, resulting in pET21α(+)_trAtKO. Wild-type AtKO was also cloned into the pET21α(+) vector, thus giving pET21α(+)_AtKO. To analyze the expression levels of trAtKO and AtKO by SDS-PAGE, pET21α(+)_AtKO, pET21α(+)_trAtKO, and the empty vector, pET21α(+), were transfected into *E. coli* BL21 (DE3). The transformants were incubated at 30 °C and 250 g in 50 mL of the LB medium containing 100 μg/mL ampicillin. When optical density at 600 nm (OD_600_) reached 0.6–0.8, 0.4 mM isopropyl *β*-d-1-thiogalactopyranoside (IPTG) was added to induce protein expression. After 3 h of induction, the cells were harvested and resuspended in 50 mM Tris–HCl (pH 6.8). The resuspended cells were disrupted by ultrasonication on ice to extract total protein. Each sample was analyzed by SDS-PAGE in a 10% (w/v) gel. Total-protein concentrations were determined by the Bradford assay.

### Deletion of *gdhA* encoding a glutamate dehydrogenase, and measurement of NADP^+^ and NADPH concentrations

An MGIKN strain with deletion of the *gdhA* gene (encoding a glutamate dehydrogenase) was constructed by deletion of *gdhA* from the genome of the MGIK strain using CRISPR/Cas9. Quantification of NADP^+^ and NADPH concentrations in strains MGIK and MGIKN was carried out with NADP/NADPH Assay Kits (Abcam, UK). Cells in the log phase were harvested, and analytes were extracted via two freeze–thaw cycles in NADP/NADPH extraction buffer.

### Construction of the artificial self-sufficient trCYP714A2-AtCPR2 fusion protein

The transmembrane region of NADPH cytochrome P450 reductase 2 of *A. thaliana* (AtCPR2) was predicted using the TMHMM program. Seventy-two amino acid residues from the N terminus of AtCPR2 were removed by PCR with specific primers (Table 2), thus yielding trAtCPR2. In a similar way, the N-terminal sequence of CYP714A2 was removed by PCR. The N terminus of trAtCPR2 was fused to the C terminus of trCYP714A2 through a flexible linker of varied length, (GGGGS)_n=1–3_, by Gibson assembly. The resulting fusion proteins were named Fusion 5, Fusion 10, and Fusion 15. The primer sequences used to construct the fusion proteins are provided in Table 2.

### Batch bioreactor fermentation

Strains MGI and MGIUK were utilized for the production of *ent*-kaurene, and the MGIUKN strain was used for the production of steviol in batch bioreactor fermentation. Seed cultures of strains MGI, MGIUK, and MGIUKN were prepared by inoculation into 4 mL of the LB medium containing appropriate antibiotics at 30 °C with shaking at 250 g for 10 h. The seed cultures were transferred to 100 mL of the TB medium containing appropriate antibiotics until OD_600_ reached 2–3; then, the precultures were transferred into a 3 L jar bioreactor (BioFlo 320, Eppendorf, USA) containing 1 L of the TB medium supplemented with 20 g/L glycerol and appropriate antibiotics; 20% (v/v) *n*-dodecane was added into fermentation medium. Fermentation was carried out at 30 °C at an air flow rate of 1.5 vvm. The dissolved-oxygen level was maintained at 30% by means of air supply or a mixture of air and pure O_2_ and via adjustment of the agitation rate between 300 and 600 rpm. pH was automatically maintained at 7.0 by the addition of 24% (v/v) NH_4_OH and 2 N HCl. Cell growth was monitored at a wavelength of 600 nm on a SPECTRAmax PLUS384 instrument (Molecular Devices, USA).

### Extraction and analysis of products

To quantify *ent*-kaurene, the *n*-dodecane layer in the bacterial culture was recovered by centrifugation, and 50 μL of the *n*-dodecane was diluted with 450 μL of ethyl acetate (EA). To extract *ent*-kaurenoic acid and steviol, fermentation broth containing cells was ultrasonicated and extracted twice with an equal volume of EA. After centrifugation for 5 min at 14,000 g, the organic phase was collected and dried in an EZ-2 *plus* centrifugal evaporator (Genevac, UK). The dried samples were dissolved in 50 μL of MeOH and then methylated with diazomethane in diethyl ether. *Ent*-kaurene, kaurenoic acid methyl ester, and steviol methyl ester were resuspended in EA and analyzed by gas chromatography with mass spectrometry (GC–MS) on Agilent 7890A, 5874C (Agilent Technologies, USA) equipped with an HP-5MS column (30 m × 0.25 mm × 0.25 μm, Agilent Technologies). The GC–MS operational conditions were as follows: initial temperature 80 °C for 1 min, ramp up to 245 °C at 15 °C/min, and ramp up to 300 °C at 5 °C/min; the flow rate of helium was 1.2 mL/min. Organic acid and glycerol concentrations in culture media were quantified using an Agilent 1200 HPLC system equipped with a refractive index detector (Agilent Technologies) and an Aminex HPX-87H column (Bio-Rad, USA) with 4 mM H_2_SO_4_ as the mobile phase. The flow rate was 0.7 mL/min, and column temperature was kept at 50 °C.

### Homology modeling and structural analysis

Fusion proteins were modeled with 10 PDB template structures by means of I-TASSER [28]. All structures were visualized in the PyMOL software (http://www.pymol.org). NADPH-binding sites of the fusion proteins were predicted with COACH [29].

## Data Availability

Not applicable.
